# A case of V180I genetic Creutzfeldt-Jakob disease presenting with conspicuous facial mimicry

**DOI:** 10.1080/19336896.2019.1651181

**Published:** 2019-08-06

**Authors:** Yasushi Iwasaki, Keiko Mori, Masumi Ito, Yoshinari Kawai

**Affiliations:** a Department of Neuropathology, Institute for Medical Science of Aging, Aichi Medical University, Nagakute, Japan; b Department of Neurology, Oyamada Memorial Spa Hospital, Yokkaichi, Japan

**Keywords:** Creutzfeldt-Jakob disease, facial mimicry, pathological laughing, startle reaction, MRI

## Abstract

Although there have been no reports of facial mimicry in patients with Creutzfeldt-Jakob disease (CJD), we encountered a patient with genetic CJD with prion protein gene codon 180 mutation (V180I gCJD) who apparently showed this interesting clinical finding. The patient was an 87-year-old Japanese woman, and the first observed CJD symptom was poor spontaneity. She gradually showed cognitive dysfunction and subsequently gait disturbance. A prion protein gene analysis revealed a V180I mutation with methionine homozygosity at codon 129. Facial mimicry was observed 7 months after disease onset and continued for approximately 9 months. Pathological laughing and startle reaction were also observed during approximately the same period, whereas myoclonus was observed at a later stage, 12 months after disease onset, and was very mild in degree. Electroencephalography studies showed a diffuse slow basic pattern without periodic sharp wave complexes. Diffusion-weighted magnetic resonance imaging showed extensive hyperintensity in the cerebral cortex, and there was also hyperintensity with edematous swelling in the same regions on T2-weighted and fluid-attenuated inversion recovery images. On the basis of the magnetic resonance imaging findings and the findings of previous case reports of V180I gCJD, we speculate that the characteristic extensive cerebrocortical involvement observed in V180I gCJD was implicated in the pathogenesis of the facial mimicry observed in this case.

## Introduction

Creutzfeldt-Jakob disease (CJD) is a fatal neurodegenerative disease that can be classified as sporadic (idiopathic), genetic (hereditary), or acquired (infectious) [1,2]. Genetic CJD (gCJD) can be further classified based on the present mutation of the prion protein (*PrP*) gene, and individual genetic mutations show variable geographic distribution and frequency [1–3]. Although most patients with sporadic CJD exhibit characteristic clinical findings including rapidly progressive cognitive impairment, myoclonus, and periodic sharp wave complexes (PSWCs) on electroencephalography (EEG), patients with gCJD frequently fail to exhibit one or more of these hallmarks depending on the type of mutation [1,2,4]. CJD with point mutation of valine to isoleucine at codon 180 of the *PrP* gene (V180I gCJD) is the most frequent gCJD form in Japan [5], whereas this variant is extremely rare in Europe and North America [3]. According to several previous reports, the clinical features of V180I gCJD are relatively uniform but significantly differ from those of typical sporadic CJD as follows [1,6–10]: (1) onset at an older age, (2) prolonged disease duration with a slower course of progression, (3) early stages of the disease characterized by cerebral cortical symptoms, such as aphasia, apraxia, and dementia, but without visual or cerebellar involvement; (4) a lower positive rate of brain-specific proteins such as neuron-specific enolase, total tau protein, and 14–3-3 protein in the cerebrospinal fluid (CSF); (5) absence of PSWCs on EEG throughout the disease course, (6) less prominent myoclonus with a later onset, and (7) rarely present family history of the disease. Furthermore, conspicuous pathological laughing and startle reaction observed in the early disease stage are also frequently present in V180I gCJD [8–10].

‘Facial mimicry’ is a clinical phenomenon pertaining to imitation of the facial movements and expressions of others [11,12]. Although, to the authors’ knowledge, there have been no reports of facial mimicry in patients with CJD, we encountered a patient with V180I gCJD conspicuously showing this finding. Clinical observations of the patient were serially investigated, and the authors speculated upon the relation between facial mimicry and the pathogenesis of V180I gCJD.

## Clinical summary

**Figure 1.**
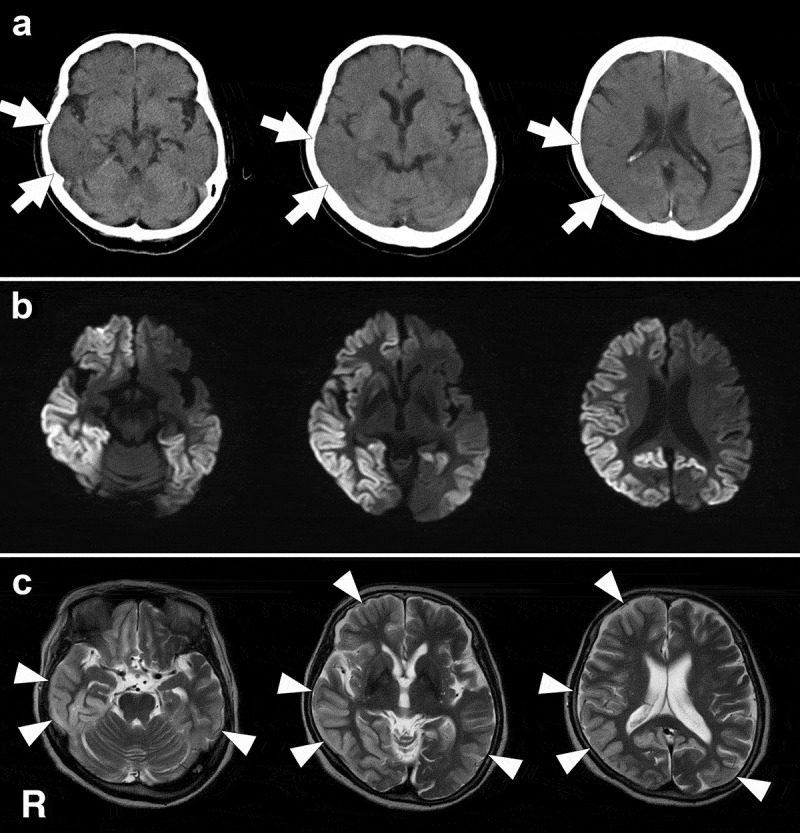
Head computed tomography (CT) and magnetic resonance imaging (MRI) findings. These images were obtained 8 months after the disease onset. Clinically, facial mimicry and pathological laughing were remarkable at this stage. a: CT images show edematous and swelling findings in the right cerebral hemisphere, particularly in the temporal and parietal lobes (arrows). In the right temporal and parietal cortices, the sulcus shows narrowing and the corticomedullary junction is generally indistinct. b: Diffusion weighted MRI shows widespread gyriform hyperintensity in the right cerebral hemisphere and left parietal and occipital lobes, except in the medial occipital regions. c: T2-weighted images show hyperintense regions with swelling (arrowheads), and the regions correspond to those that were hyperintense on diffusion weighted MRI. R, right side.

**Figure 2.**
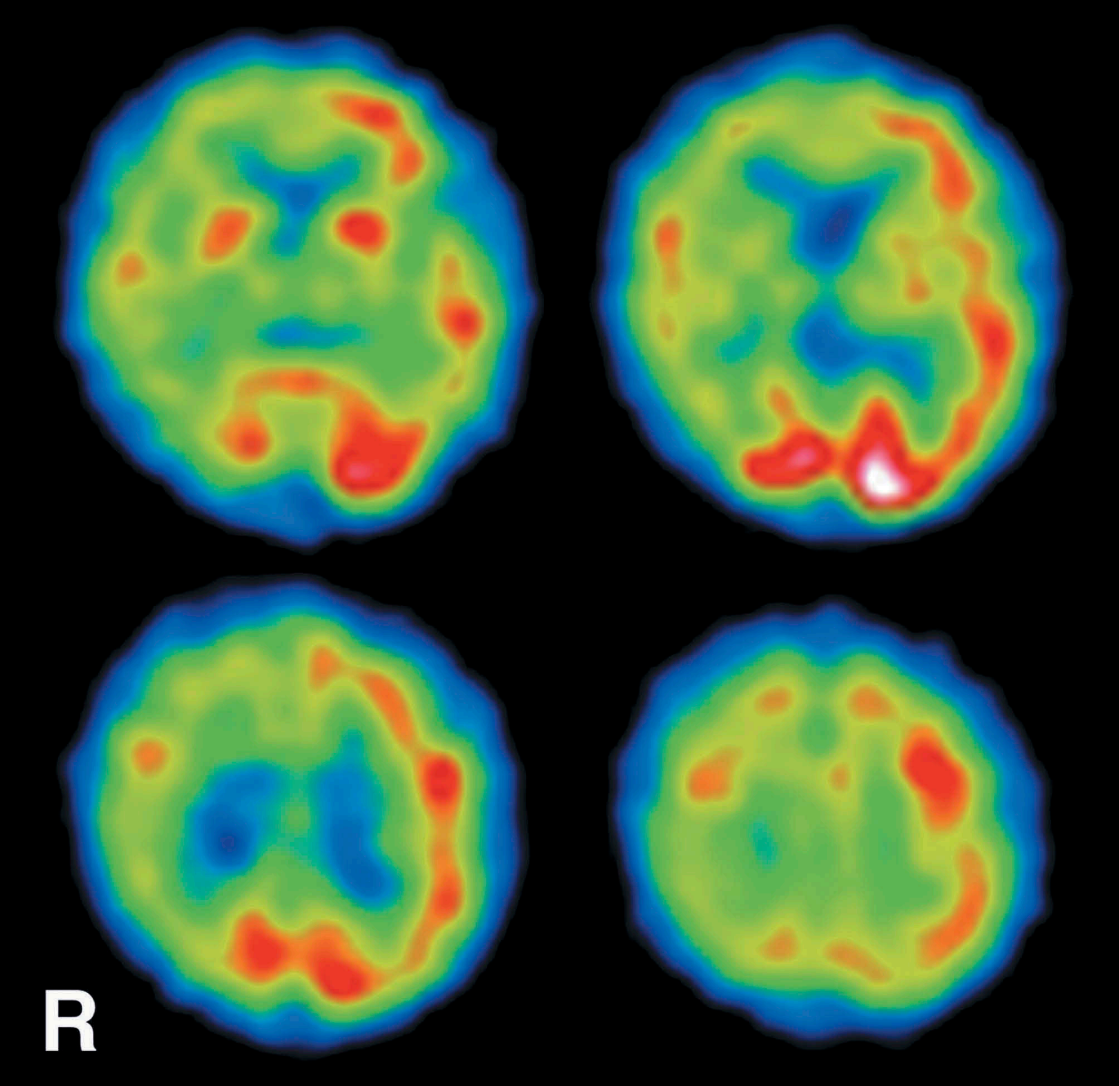
SPECT findings. ^99m^Tc-ECD-SPECT images obtained 5 months after the onset of symptoms revealed decreased regional cerebral blood flow in the right cerebral hemisphere, particularly in the parietal lobe. The regions with decreased cerebral blood flow almost corresponded to the regions that were hyperintense on diffusion weighted magnetic resonance imaging. ^99m^Tc-ECD-SPECT, technetium-99m ethyl cysteinate dimer single photon emission computed tomography; R, right side.

**Figure 3.**
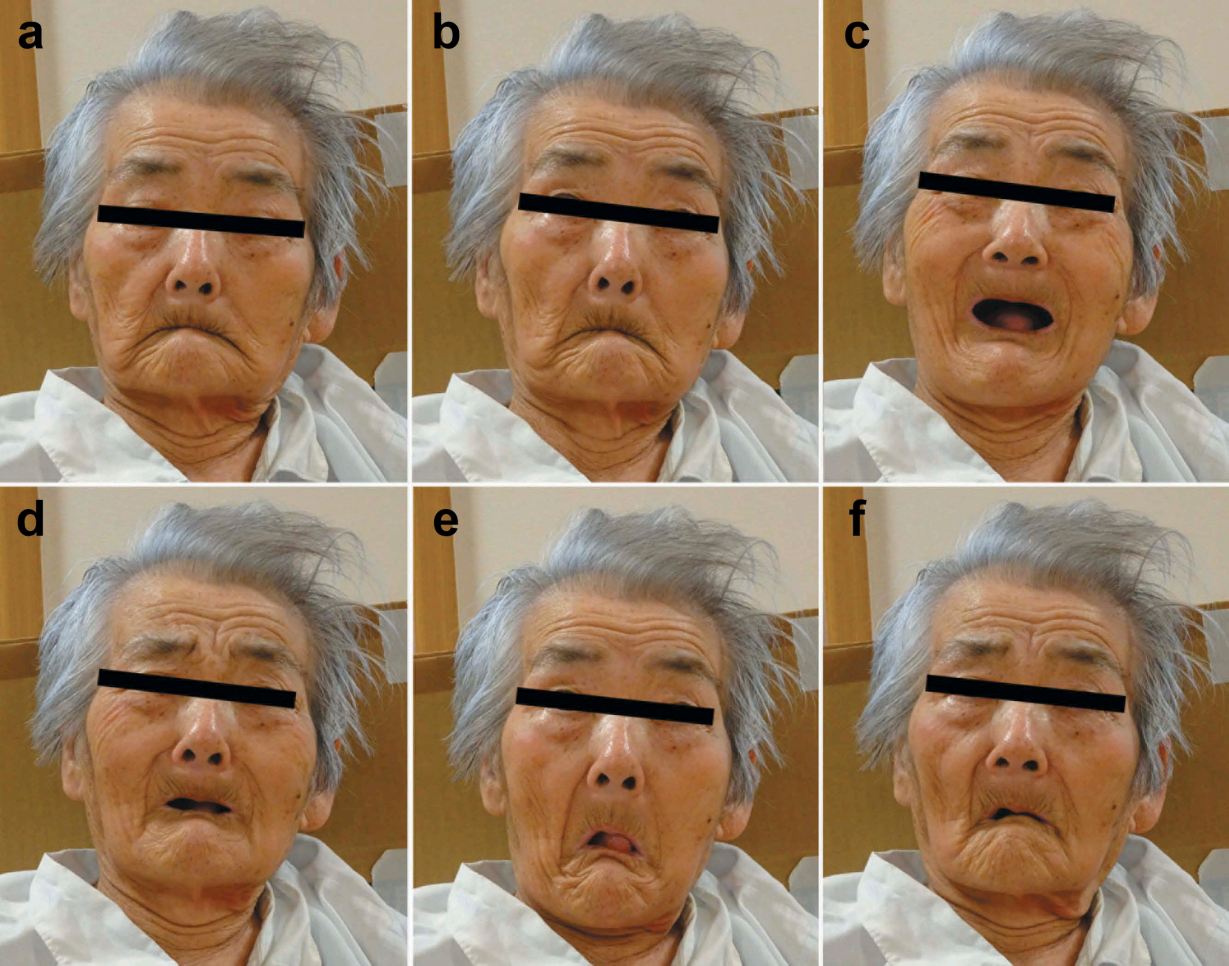
Expression of facial mimicry of the patient. These photographs were obtained 8 months after disease onset. The physician faced the patient and sequentially showed several facial expressions to the patient. The physician gazed at the patient (a). Then, sequentially, the physician widely opened the eyes (b) and showed a smiling expression (joy) (c), sadness (tragedy) (d), astonishment (surprise) (e), and a wry expression (grimacing)(f).

The family of an 87-year-old Japanese woman noticed that she had developed poor spontaneity and that she generally showed decrease of interest in her daily activities and cognitive dysfunction, particularly memory disturbance. Two months after symptom onset, soliloquy and night delirium were observed. The following month, she showed gait disturbance with falls and her posture inclined to the left during walking. Her activities of daily living and disorientation progressively worsened; she could not recognize her room in the house, and assistance became necessary for dressing. She was referred to the Department of Neurology 6 months after onset of symptoms because she became unable to walk without assistance. She had no known family history of prion disease and no past history of neuropsychiatric disorder. She was undergoing treatment for diabetes. Neurological examination revealed hypertonia in her extremities, with left-side predominance. The deep tendon reflex of the right-side extremities was mildly brisk. Her consciousness was preserved, but conversation was not fluent; simple conversation was still possible. Palmomental, sucking, and snout reflexes were not recognized. Head computed tomography showed swelling in the right cerebral hemisphere (Figure 1a). Diffusion-weighted (DW) magnetic resonance imaging (MRI) showed extensive gyriform hyperintensity of the cerebral cortex (Figure 1b). The hyperintense regions on DW imaging (DWI) were also hyperintense with edematous swelling on T2-weighted (Figure 1c) and fluid-attenuated inversion recovery (FLAIR) images and showed cerebral blood flow decrease on single photon emission computed tomography (Figure 2). Semi-quantitative CSF analysis revealed positive findings of 14–3-3 protein and total tau protein, but the real-time quaking-induced conversion test result was negative. A PrP gene analysis using genomic DNA extracted from peripheral blood revealed a V180I mutation with methionine homozygosity at codon 129 and glutamic acid homozygosity at codon 219. EEG studies were performed several times and they consistently showed a diffuse slow basic pattern without PSWCs. Pathological laughing and startle reaction appeared 7 months after symptom onset, and subsequently, she showed facial mimicry; she imitated the facial expressions of the person who faced her, including members of her family and of the medical staff, such as sadness, wide opening of the eyes or mouth, surprise, smiling, tearful expressions, and grimaces (Figure 3). However, she did not imitate the movements of extremities of persons, and utilization behavior was not observed. Although daily conversation had already become impossible at the time this finding was observed, she had not yet reached the akinetic mutism state. In addition, she did not experience impairments of consciousness, such as delirium or stupor. Myoclonus was observed 12 months after symptom onset, but the degree was generally mild. The facial mimicry and pathological laughing were remarkable for several months and gradually disappeared along with the startle reaction 16 months after disease onset. Although the patient gradually reached the akinetic mutism state 18 months after disease onset, at the time of writing, she could still intake food orally with assistance. The patient was still alive at the age of 89 years, 22 months after symptom onset.

## Discussion

In interpersonal encounters, individuals often spontaneously change their facial expressions in response to the emotional expressions of other persons; such changes are referred to as facial mimicry (alternatively referred to as facial imitation or facial mimicking), and they are generally considered a reflex-like automatic response beyond intentional control [11,12]. Facial mimicry is also physiologically observed in neonates, in which case, it is particularly referred to as neonatal imitation (alternatively referred to as infant imitation or newborn imitation) [13]; these findings from the early developmental stages also suggest that facial mimicry occurs automatically as a primitive reaction [12,13]. The occurrence of facial mimicry has also been shown in many non-human mammals, including apes [14,15], monkeys [16], and dogs [17].

Facial mimicry is modulated by various factors, i.e., attention deployment and sensitivity, detection of valence and interest, emotions, and social motivations [11], and its pathological background is suspected to involve the mirror neuron system [18,19]. Mirror neurons, a type of sensory-motor cell, are activated when an individual performs an action or observes another individual performing the same action and are of interest in the study of certain social behaviors, such as empathy and imitation [18,19]. Neuroanatomically, mirror neurons have been found in areas of the inferior frontal cortex (ventral premotor cortex) and the inferior parietal lobule [18,20]. These two regions work in concert and receive input from areas involved in the perceptual processing of biological movement, such as the areas of the superior temporal sulcus [18,20]; the pars opercularis of the inferior frontal cortex is suspected to be a particularly important component of the mirror neuron system [18,20]. Interestingly, functional brain imaging to examine brain activity during mimicry of facial expressions and related regional responses to the magnitude of expression-induced facial movements also showed that the inferior frontal gyrus has a central role in the imitation of emotional expressions [21]. Based on these reports, we speculate that the appearance of facial mimicry in the present case indicated that the mirror neurons were functionally preserved, at least during the period when the symptom was observed.

Based on the finding of facial mimicry in the present case, we speculate that the function of both visual recognition, including from the optic nerve to the primary visual cortex in the occipital cortex, and facial movement, including the facial nerve nucleus and primary motor cortex in the precentral gyrus, were preserved, at least during the period that the symptom was observed. According to previous autopsy case reports of V180I gCJD, the neurons of the cerebral neocortex in such cases show a tendency toward preservation despite the extensive severe spongiform changes and long disease duration [1,6,7,9,10]. Furthermore, the precentral gyrus and in particular the medial portion of the occipital lobe tend to be pathologically preserved in patients with V180I gCJD [1,9,10]. We believe that these pathological observations appear to support our hypothesis.

Based on the timing of symptom appearance, we also speculate that the pathogenesis of the facial mimicry observed in this case may have been associated with, at least in part, the pathological laughing and startle reaction because these findings were observed for approximately the same period and continued for several months. Regarding pathological laughing, the symptom is frequently recognized in patients with V180I gCJD who have not yet reached the akinetic mutism state and it has not been reported in other subtypes of CJD [8–10]. In cases of V180I gCJD, pathological laughing is usually observed together with the startle reaction and for almost the same period, and the degree of severity of both symptoms is almost parallel during the observation period [8,9]. The dissociation between conspicuous pathological laughing and the startle reaction and less prominent myoclonus is considered to be significant when considering the pathogenesis of V180I gCJD [1,8–10].

The MRI findings in V180I gCJD are very peculiar, and we can estimate a diagnosis of V180I gCJD when special presentations are recognized; the findings involve extensive cerebral cortical hyperintense regions on DWI with edematous swollen presentation on T2-weighted and FLAIR images [6,8–10,22]. Furthermore, peculiar findings are characteristically absent in the medial occipital cortices in the early disease stage [10,22]. The MRI findings are fundamentally well matched with the pathological findings in V180I gCJD [1,9,10]. On the basis of MRI and autopsy findings, we previously speculated that pathological laughing is induced by extensive cerebrocortical involvement without apparent brainstem involvement, which is characteristic of V180I gCJD [8–10], and we now surely speculate that facial mimicry is also related to these MRI and pathological findings.

Based on the several findings described above, we suggest that facial mimicry may be a comparatively frequent observation in patients with V180I gCJD; however, to our knowledge, it has not been previously observed. The number of reported V180I gCJD cases is small and limited; therefore, evaluation of additional cases will be necessary in order to confirm our observations and speculations regarding facial mimicry in V180I gCJD.
